# Chemical Composition, Anti-Quorum Sensing, Enzyme Inhibitory, and Antioxidant Properties of Phenolic Extracts of *Clinopodium nepeta* L. Kuntze

**DOI:** 10.3390/plants10091955

**Published:** 2021-09-18

**Authors:** Hatem Beddiar, Sameh Boudiba, Merzoug Benahmed, Alfred Ngenge Tamfu, Özgür Ceylan, Karima Hanini, Selcuk Kucukaydin, Abdelhakim Elomri, Chawki Bensouici, Hocine Laouer, Salah Akkal, Louiza Boudiba, Rodica Mihaela Dinica

**Affiliations:** 1 Laboratory of Organic Materials and Heterochemistry, Tebessa University, Constantine Road, Tebessa 12002, Algeria; hatem.beddiar@univ-tebessa.dz (H.B.); merzoug.benahamed@univ-tebessa.dz (M.B.); 2 Laboratory of Bioactive Molecules and Applications, Tebessa University, Constantine Road, Tebessa 12002, Algeria; sameh.boudiba@univ-tebessa.dz (S.B.); karima.hanini@univ-tebessa.dz (K.H.); 3 Department of Chemical Engineering, School of Chemical Engineering and Mineral Industries, University of Ngaoundere, Ngaoundere 454, Cameroon; 4 Food Quality Control and Analysis Program, Ula Ali Kocman Vocational School, Mugla Sitki Kocman University, Mugla 48147, Turkey; ozgurceylan@mu.edu.tr; 5 Department of Medical Services and Techniques, Koycegiz Vocational School of Health Services, Mugla Sitki Kocman University, Mugla 48800, Turkey; selcukkucukaydin@gmail.com; 6 CNRS, COBRA (UMR 6014), Normandie University, UNIROUEN, INSA Rouen, 76000 Rouen, France; hakim.elomri@univ-rouen.fr; 7 Research Center on Biotechnology, Ali Mendjli New City UV 03, BP E73, Constantine 25000, Algeria; chawkiislam@yahoo.fr; 8 Laboratory for the Valorization of Natural Biological Resources, Ferhat Abbas University, UFA-Setif 1, Setif 19000, Algeria; hocine_laouer@yahoo.fr; 9 Laboratory of Phytochemistry, Physicochemical and Biological Analyses, Mentouri University, Ain El Bey Road, Constantine 25000, Algeria; salah62dz@gmail.com; 10 Laboratory of Water and Environment, Tebessa University, Constantine Road, Tebessa 12002, Algeria; boudiba.louiza@univ-tebessa.dz; 11 Department of Chemistry, Physics and Environment, Faculty of Sciences and Environment, Dunarea de Jos University, 47 Domneasca Str., 800008 Galati, Romania

**Keywords:** *C. nepeta*, phenolic compounds, antioxidant, antimicrobial, anti-quorum sensing, enzyme inhibition, Alzheimer’s disease

## Abstract

Phenolic extracts of *Clinopodium nepeta* were prepared and their preliminary phenolic profiles determined using HPLC-DAD with 26 phenolic standards. Apigenin (21.75 ± 0.41 µg/g), myricetin (72.58 ± 0.57 µg/g), and rosmarinic acid (88.51 ± 0.55 µg/g) were the most abundant compounds in DCM (dichloromethane), AcOEt (ethyl acetate), and BuOH (butanol) extracts, respectively. The DCM and AcOEt extracts inhibited quorum-sensing mediated violacein production by *C. violaceum* CV12472. Anti-quorum-sensing zones on *C. violaceum* CV026 at MIC (minimal inhibitory concentration) were 10.3 ± 0.8 mm for DCM extract and 12.0 ± 0.5 mm for AcOEt extract. Extracts showed concentration-dependent inhibition of swarming motility on flagellated *P. aeruginosa* PA01 and at the highest test concentration of 100 μg/mL, AcOEt (35.42 ± 1.00%) extract displayed the best activity. FRAP assay indicated that the BuOH extract (A_0.50_ = 17.42 ± 0.25 µg/mL) was more active than standard α-tocopherol (A_0.50_ = 34.93 ± 2.38 µg/mL). BuOH extract was more active than other extracts except in the ABTS^●+^, where the DCM extract was most active. This antioxidant activity could be attributed to the phenolic compounds detected. *C. nepeta* extracts showed moderate inhibition on acetylcholinesterase (AChE), butyrylcholinesterase (BChE), tyrosinase, and α-amylase. The results indicate that *C. nepeta* is a potent source of natural antioxidants that could be used in managing microbial resistance and Alzheimer′s disease.

## 1. Introduction

Throughout the ages, human beings have depended on plants for their basic needs such as source of food [1], medicine [2,3], carpentry [4], fertilizer [5,6], and aromatics [7], amongst others. The use of this rich source of natural compounds and their derivatives is increasing day by day and continuous progress is being made in the development of proper extractive methods. Additionally, there is much progress and advancements in the methods of characterization and identification of valuable phytoconstituents contained in the various plant extracts as well as their versatile eco-friendly applications, in particular the discovery of new and safer therapeutic agents with minor side effects [8,9]. Because of their richness in a large variety of secondary plant metabolites, such as flavonoids, alkaloids, terpenoids, tannins, etc. [10], the extracts are capable of possessing significant and numerous bioactivities which makes them useful in traditional medicine [11,12,13,14,15]. It is worth mentioning that despite the increasing usage of plant extracts for medical purposes, a majority of the medicinal plant extracts and their chemical constituents are yet to be identified and some of them have been evaluated for their biological activities only [16,17]. Medicinal plants and natural products have become an attraction for modern scientific research involving an intersection of phytochemistry and pharmacology due to the diversified health benefits they possess. Nowadays, a few of them have been tested and approved as safe chemotherapy drugs with minor side effects (vinblastine, rubomycine, colchamine, vincristine, etc.) [18,19,20,21]. Others have been proven to act as natural antioxidants [22], motivating researchers to focus on new nontoxic alternatives, to substitute the unstable and toxic synthetic ones [23,24,25]. Many medicinal plants are rich in phenolic compounds. Phenolic compounds (flavonoids, phenolic acids, etc.), as an inexhaustible source of scientific research represent a good example, due to their widespread groups of metabolites, known for benefits to human health and they have been a bottomless source of scientific research [26]. These compounds are produced in plants to counteract UV radiation, desiccation, thermal shock, pathogenic predators, competitive species, and to attract pollinators [27]. HPLC-DAD (High-Performance Liquid Chromatography Diode Array Detector) analysis is one of the common methods used for the identification of the phenolic compounds. Due to the biological activities (antibacterial, antifungal, antiviral, and antioxidant) of phenolic compounds, they have a significant role in some metabolic pathways [28,29,30].

*Clinopodium* (Lamiaceae) is a genus composed of about 135 flowering species, largely dispersed in southern and southeastern Europe and over the Mediterranean, North and Latin America right up to western Asia [31,32]. This genus is commonly rich in phenolic compounds and essential oils [31,32,33]. Species of this genus have exhibited many biological activities such as antioxidant, antibacterial, antifungal, antiviral, and anti-inflammatory [34,35,36,37]. Sarikurkcu et al. revealed the isolation of protocatechuic acid, (+)-catechin, chlorogenic acid, caffeic acid, ferulic acid, rosmarinic acid, and apigenin from *Clinopodium vulgare*. Moreover, its extracts showed antioxidant and enzymatic inhibition properties [38]. Recently, a study established on *Clinopodium nepeta* essential oil showed antifungal and insecticidal properties [39]. However, no previous works were found concerning the phenolic compounds of this plant.

The purpose of this study was to determine the preliminary phenolic composition and in vitro biological activities such as antioxidant, anticholinesterase, anti-tyrosinase, α-amylase inhibitory, and anti-quorum-sensing activities of dicholomethane, ethyl acetate, and butanol extracts of *C. nepeta.*

## 2. Results

### 2.1. HPLC-DAD Phenolic Composition

**Figure 1:**
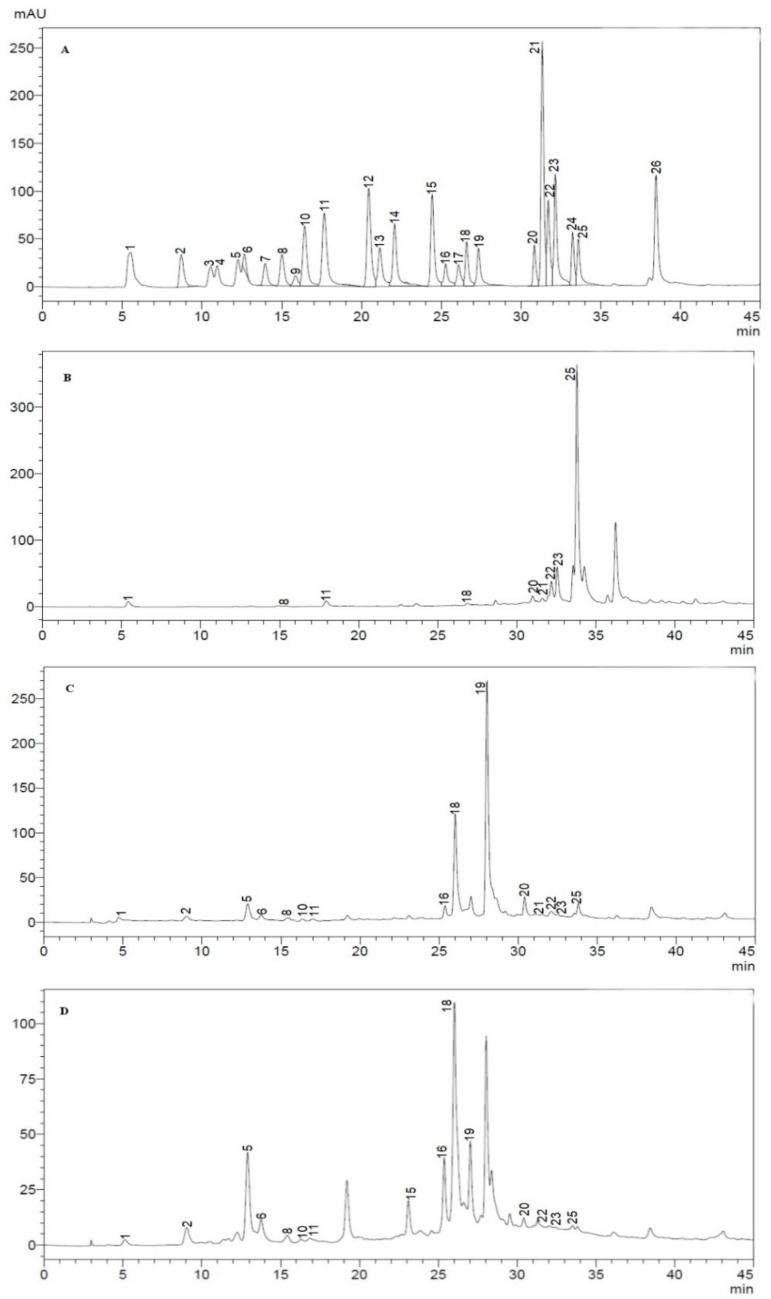
HPLC chromatograms of phenolic compounds; (**A**): Standards, (**B**): DCM extract of *C. nepeta*, (**C**): AcOEt extract of *C. nepeta*, (**D**): BuOH extract of *C. nepeta.*

Phenolic compounds are widespread in the plant kingdom, and various solvents including ethyl acetate (AcOEt), n-butanol (BuOH), and dichloromethane (DCM) have been used to extract them from *C. nepeta* in this study. The extracts were characterized using HPLC-DAD and are reported in Table 1. These results confirm that these solvents are effective in the extraction process since the number of phenolic compounds detected and quantified is appreciable. The chromatograms of the 26 standard phenolic compounds used in identification and quantification as well as those detected in each extract are provided in Figure 1. Apigenin was the compound most abundant in the DCM extract (21.75 ± 0.41 µg/g), myricetin in the AcOEt extract (72.58 ± 0.57 µg/g), and rosmarinic acid in the BuOH extract (88.51 ± 0.55 µg/g). Nine phenolic compounds (rosmarinic acid, myricetin, quercetin, luteolin, hesperetin, apigenin, vanillic acid, vanillin, and gallic acid) were found in all extracts, though in different amounts. However, *trans*-cinnamic acid was present only in the DCM extract (4.21 ± 0.35 µg/g) and AcOEt extract (5.28 ± 0.11 µg/g). Protocatechuic acid (DCM extract = 8.24 ± 0.25 µg/g and AcOEt extract = 8.83 ± 0.15 µg/g), chlorogenic acid (DCM extract = 18.79 ± 0.17 µg/g and AcOEt extract = 30.56 ± 0.34 µg/g), *p*-hydroxy benzoic acid (DCM extract = 14.48 ± 0.26 µg/g and AcOEt extract = 16.25 ± 0.13 µg/g), syringic acid (DCM extract = 1.50 ± 0.12 µg/g and AcOEt extract = 2.33 ± 0.10 µg/g), rutin (DCM extract = 5.83 ± 0.18 µg/g and AcOEt extract = 12.20 ± 0.25 µg/g), myricetin (DCM extract = 72.58 ± 0.57 µg/g and AcOEt extract = 52.40 ± 0.36 µg/g), kaempferol (7.40 ± 0.18 µg/g), and coumarin (10.45 ± 0.23 µg/g) were detected and quantified exclusively in the BuOH extract.

### 2.2. Violacein Inhibition Percentage and Quorum-Sensing Inhibition

The eventual quorum-sensing inhibition of the plant extracts were evaluated by using *C. violaceum* CV12472 bacteria which produces purple violacein when growing and *C. violaceum* CV026, a mutant strain that does not produce violacein unless an external acylhomoserine lactone (AHL) is supplied to it. The minimal inhibitory concentration (MIC) that is the lowest concentration at which there was no visible growth of the bacteria, inhibited by the extracts, was evaluated, and the violacein inhibition and quorum-sensing (QS) assays were done at MIC and sub-MIC. The MIC values in mg/mL were 0.5 (DCM extract), 1 for both AcOEt and BuOH extracts on *C. violaceum* CV12472 and on *C. violaceum* CV026. MIC values in mg/mL (Table 2) were 0.5 (DCM extract), 0.625 (AcOEt extract), and 0.625 (BuOH extract). At MIC, only DCM extract (100.0 ± 0.0%) and AcOEt extract (18.9 ± 4.1%) inhibited violacein production in *C. violaceum* CV12472. At MIC/2 percentage inhibitions of violacein production were 19.2 ± 1.1% and 4.5 ± 0.3% for DCM and AcOEt extracts, respectively, and only DCM extract inhibited violacein production at MIC/4 (9.0 ± 0.5%). Anti-quorum-sensing zones measured in millimeters on *C. violaceum* CV026 plates were 10.3 ± 0.8 mm and 12.0 ± 0.5 mm for DCM and AcOEt extracts, respectively. The BuOH extract did not show any activity in these assays.

### 2.3. Swarming Motility Inhibition of C. nepeta Extracts on P. aeruginosa PA01

Swarming movement is used by flagellated bacteria, such as *P. aeruginosa*, to move on surfaces. The ability of *C. nepeta* extracts to inhibit this swarming movement on *P. aeruginosa* was evaluated and is presented in Table 3. The inhibition percentage of swarming motility was determined at 100 14.89 ± 0.40 µg/mL, 35.42 ± 1.00 µg/mL, and 08.27 ± 3.11 µg/mL for DCM, AcOEt, and BuOH extracts, respectively. Only the AcOEt extract was able to inhibit swarming at 75 µg/mL (17.95 ± 2.01%) and 50 µg/mL (7.60 ± 0.33%).

### 2.4. Enzyme Inhibitory Activities of C. nepeta Extracts

The extracts’ ability to inhibit AChE, BChE, tyrosinase, and α-amylase were evaluated and are reported in Table 4. The maximum test concentration was 200 µg/mL, and samples that did not show up to 50% inhibition within this range have their IC_50_ values reported as greater than 200 µg/mL. In the AChE assay, the IC_50_ value of DCM extract was 170.1 ± 1.58 µg/mL compared to galantamine’s 5.50 ± 0.18 µg/mL while the other extracts had IC_50_ values > 200 µg/mL. In the BChE assay, DCM and AcOEt extracts had IC_50_ values of 73.06 ± 0.83 and 187.8 ± 1.57 µg/mL, respectively, while galantamine showed IC_50_ of 42.38 ± 0.50 µg/mL. Kojic acid was used as standard and exhibited in anti-tyrosinase assay an IC_50_ value of 23.80 ± 0.25 µg/mL while all extracts had IC_50_ values > 200 µg/mL. The extracts showed inhibition on α-amylase though IC_50_ values were > 200 µg/mL, but still moderate as compared to acarbose with IC_50_ of 365.93 ± 10.70 µg/mL. It should be noted that these activities were moderate because when IC_50_ values were above the highest test concentration of 200 µg/mL, the extracts’ percentage inhibitions remained appreciable and were an indication of a good potential with percentage inhibitions which are very close to 50% in some extracts.

### 2.5. Antioxidant Activity of C. nepeta Extracts

Substances that can quench free radicals and reactive oxygen species, especially in the human system, are referred to as antioxidants, and their effects reduce the risk of many disorders. The antioxidant effect of medicinal plants extracts can be evaluated through various methods. In this study, DPPH^●^ (2,2-diphenyl-1-picrylhydrazyl radical), ABTS^●+^ (2,2′-azino-bis(3-ethylbenzothiazoline-6-sulfonic acid radical cation)), GOR (galvinoxyl radical), CUPRAC (cupric reducing antioxidant capacity), phenanthroline, and FRAP (ferric reducing antioxidant power) assays were used and the results given on Table 5. The concentration at which 50% inhibition was observed, given as IC_50_ values, was determined in the DPPH^●^, ABTS^●+^, and GOR assay. In the CUPRAC, phenanthroline, and FRAP assays, the results were given as A_0.50_ corresponding the concentration at 0.5000 absorbance. In the DPPH^●^ radical scavenging assay, the extract in BuOH was the most active extract with an IC_50_ value of 8.12 ± 0.11 µg/mL as compared to BHA (butylated hydroxyanisole) with an IC_50_ value of 5.73 ± 0.41 µg/mL. In the ABTS^●+^ assay, the DCM extract showed the best activity with an IC_50_ value of 9.56 ± 1.12 µg/mL relatively close to that of the standards (reference antioxidant compounds) BHA (IC_50_ = 1.03 ± 0.00 µg/mL) and BHT (butylated hydroxytoluene) (IC_50_ = 1.59 ± 0.03 µg/mL). In the GOR assay, the BuOH extract was the most active and exhibited an IC_50_ value of 10.07 ± 0.40 µg/mL, while the standards BHA and BHT had IC_50_ values of 5.38 ±0.06 and 3.32 ± 0.18 µg/mL, respectively. In the radical scavenging assays (DPPH^●^, ABTS^●+^, and GOR), the extracts showed appreciable activity though not more active than the standards, and the BuOH extract was most active except ABTS^●+^, where the DCM extract was most active. Apart from these antiradical assays, three other methods were used. In the CUPRAC assay, the BuOH extract was most active with A_0.50_ of 29.44 ± 0.65 µg/mL, and the standards BHA and BHT had A_0.50_ values of 3.64 ± 0.19 and 9.62 ± 0.87 µg/mL, respectively. As concerns the phenanthroline assay, the BuOH extract had the highest activity amongst the extracts with an A_0.50_ of 9.85 ± 0.07 µg/mL compared to BHA and BHT (0.93 ± 0.07 and 2.24 ± 0.17 µg/mL, respectively). The measurement of FRAP indicated that the BuOH extract (A_0.50_ = 17.42 ± 0.25 µg/mL) was more active than α-tocopherol (A_0.50_ = 34.93 ± 2.38 µg/mL) used as standard, but the ascorbic acid (A_0.50_ = 6.77 ± 1.15 µg/mL) was most active.

## 3. Discussion

Within the Algerian flora, phenolic compounds are ubiquitous and one of the most abundant classes of natural compounds in various plant species [40]. Though *C. nepeta* has been studied for its essential oil composition, the phenolic content of this Algerian plant has not yet been revealed. The results of preliminary phenolic chemical composition reported here indicate that DCM, AcOEt, and BuOH extracts of *C. nepeta* contain various selected phenolic compounds. High-performance liquid chromatography with photodiode array detection (HPLC-DAD) against 26 standard phenolic compounds was used to identify and quantify individual phenolic compounds [41]. Several classes of phenolic organic compounds exists, such as phenolic acids, tannins, flavonoids, lignans, and stilbenes, and the common feature amongst the compounds is the presence of aromatic ring with one or more hydroxyl substituents in their chemical structures [40,41,42]. The phenolic compounds were detected in different amounts in each of the extracts and they can be classified into different structural subgroups. It can be observed that the number and quantities of phenolic compounds in each extract increased with the polarity of the extraction solvent [43,44]. The most polar extract (BuOH extract) contains more phenolic compounds than the AcOEt and the DCM extracts [43,44]. A careful selection of extraction methods and solvents would allow maximum depletion of the target components contained in plants and avoid their chemical or biological alteration. In one study, RP-HPLC-DAD using seven phenolic standards, benzoic acid was detected in *Clinopodium nepeta*, collected in Turkey and *p*-coumaric acid was absent [45]. In this study, several phenolic standards were used and 3-Hydroxy benzoic acid and *p*-Hydroxy benzoic acid were detected, and *p*-coumaric acid was absent. *p*-Hydroxy benzoic and quercetin were absent in the *C. nepeta* from Turkey, while they were detected in *C. nepeta* from Algeria. These differences can be attributed to environmental factors (biotic and abiotic), age, cultivar, climatic factors, as well as the plant parts, extraction methods, and conditions. Phenolic compounds have gained much attention because of their numerous health benefits and biological activities.

The effect of *C. nepeta* extracts on quorum-sensing (QS) mediated processes such as inhibition of violacein production in *C. violaceum* CV12472 and *C. violaceum* CV026, showed that this plant could not only kill bacteria but also can reduce their severity and eliminate their resistance by disrupting QS networks. Antibiotics based only on bacterial growth inhibition or bactericidal effects are problematic because of development of resistance strains that posing health problems [46,47,48]. The BuOH extract contained many phenolic compounds but showed neither violacein inhibition nor anti-QS activity. This indicates that QS inhibitory compounds could be extracted from *C. nepeta* by less polar solvents. Since only DCM and AcOEt extracts inhibited violacein formation and QS, the activity could be attributed to some phenolic compound detected exclusively in these two extracts, such as *trans*-cinnamic acid, but this will require further investigation with authentic sample of the compounds. This plant has been shown to possess antimicrobial activity [45]; however, its anti-QS effect is reported here for the first time. The extracts inhibited swarming motility in *P. aeruginosa* PA01. During bacterial communication, signal molecules can diffuse within colonies and regulate the motility of microorganisms, and swarming movement is implicated in QS-mediated biofilm formation [28,46]. In adversity such as antibiotics and starvation, most microorganisms constitute self-organized biofilm which protects their communities permitting them to remain viable despite adverse conditions [30,49]. This accounts for resistance and severity during infections, therefore, the extracts of *C. nepeta* can be used in reducing microbial resistance and the severity of the infections. This potential activity can be attributed to the chemical constituents contained in the various extracts amongst which are the selected phenolic compounds identified in the extracts. The *C. nepeta* extracts inhibited swarming motility which is a process used by bacteria prior to biofilm formation. The inhibition of swarming motility mostly in flagellated bacteria can reduce the risk of biofilm formation. There is rising interest in swarm motility which is used to colonize solid surfaces and many factors can define it in various bacterial systems such as the secretion of a surfactant to reduce surface tension and allow spreading, increasing in the number of flagella per cell and promoting movement in multicellular groups rather than as individuals [50].

All extracts exhibited inhibitory potentials on cholinesterase, tyrosinase, and α-amylase enzymes. These enzymes which are key enzymes were intervening in some human pathologies such as diabetes for α-amylase, neurodegenerative disorders such as Parkinson’s disease for tyrosinase and Alzheimer’s disease for AChE and BChE [51]. The positive controls used in the cholinesterases, tyrosinase, and α-amylase assays were galantamine, kojic acid, and acarbose, respectively. In these enzyme inhibitory assays, the standards were more active than the extracts of *C. nepeta*. Amongst the extracts, the DCM extract was the most active in AChE, BChE, and tyrosinase inhibitory activities assay, while the BuOH extract had highest activity in the α-amylase inhibitory assay. Amongst the phenolic compounds detected in these extracts are some bioactive ones which have been proven to possess anticholinesterase activity and can equally inhibit other enzymes [52]. Since the extracts can inhibit α-amylase, it means that they can be used in managing diabetes by reducing postprandial glucose levels. The enzyme α-amylase hydrolyses carbohydrates into sugars, and inhibition of this enzyme is a useful strategy to decrease blood glucose levels and diabetic conditions [53]. Cholinesterase inhibitors provide a suitable and effective means of treating cognitive symptoms of neurological disorders [54]. The ability of the plants to inhibit cholinesterase enzymes indicates its potential use in remedying Alzheimer’s disease.

Oxidative stress is an important determinant of diabetes-related complications, as the overproduction of reactive oxygen species is associated with hyperglycemia and can likely lead to an imbalance between the quantity of antioxidants inside the body and free radicals which is a condition that is related to hyperglycemia [55]. The evaluation of comprehensive potential antioxidant activity of plant extracts is required to be done through multiple and complementary methods since plant extracts consist of complex phytochemicals that can undergo various oxidative processes. The antioxidant effect of *C. nepeta* extracts was evaluated by six methods where the BuOH extract was the most active. The results are significant (*p* < 0.05) as they were close to those of the various standard antioxidants used in the assay. The BuOH extract was the most active in all assays except in the ABTS^+•^ one, where the most active sample was the DCM extract. The anti-free radical activities of ABTS^+•^, DPPH^•^, and galvinoxyl as well as the CUPRAC, Phenanthroline, and FRAP could be attributed to phenolic compounds. Protection against certain diseases is the most important property of antioxidants, such as phenolic compounds, which protect cells against damage caused by free radicals by combining them to create stable, neutral, and harmless compounds [56]. Naturally occurring polyphenols are expected to reduce the risk of various life-threatening diseases, including cardiovascular diseases and cancer, due to their antioxidant activity, the reason for which the study of antioxidant compounds from foods and medicinal natural sources has gained increased significance [57]. The results here suggest that phenolic compounds contained in *C. nepeta* extracts may exert antioxidant properties, as free radical scavengers, as source of hydrogen, or as oxygen singlet extinguishers and chelating agents of metal ions. The good antioxidant activity observed for *C. nepeta* extracts corroborates previous studies [45]. Utilization and development of more effective antioxidants of natural medicinal plants are desired as these antioxidants are considered safer than synthetic ones [58,59].

The difference in activity between the extracts of the investigated plant can be explained by the complex chemical composition of phenolic compounds contained in it in varying amounts and which could act in synergy to generate different behaviors.

## 4. Materials and Methods

### 4.1. Plant Material

The aerial parts of *Clinopodium nepeta* were collected from the Annaba Region of Algeria during its flowering period in October 2018. The plant was identified by Dr. Houcin LAOUAR of the Laboratory for the Valorization of Natural Biological Resources, Ferhat Abbas University, Setif 1, Algeria. The plant was authenticated as *Clinopodium nepeta* L. Kuntze according to the French botanist Errol Vella, from Montpellier under the voucher specimen number LVBN/38/2021.

### 4.2. Extraction

The plant material was dried in shade, and then ground. A total of 500 g of dried plant material was extracted with a methyl alcohol:water (7:3) solution at room temperature. The mixture was filtered after 48 h and concentrated on a rotavapor under reduced pressure to obtain a crude extract in form of a solid paste. The crude extract was after dissolved in water, filtered, and further partitioned using liquid–liquid extraction with organic solvents, ethyl acetate (AcOEt), dichloromethane (DCM), and n-butanol (BuOH) in order of increasing polarity. The separated fractions of dichloromethane (DCM), ethyl acetate (AcOEt), and n-butanol (BuOH) were evaporated to dryness to give the crude extracts used later in the analysis in different concentrations [60].

### 4.3. Determination of Phenolic Compounds

To identify the phenolic compounds, the HPLC-DAD method was used. For this analysis, the plant extracts were solubilized in a mixture of water–methanol (80:20). After, the sample was filtered through a disposable LC filter disk of 0.20 μm and separated on an Inertsil ODS-3 reverse phase C18 column [61,62]. The flow rate was 1.0 mL/min and 20 μL was the injection volume of the sample. Mobile phase A consisted of 0.5% acetic acid in water and the mobile phase B was formed of 0.5% acetic acid in methanol. The gradient was realized as follows: 0–10% B (0–0.01 min); 10–20% B (0.01–5 min); 20–30% B (5–15 min); 30–50% B (15–25 min); 50–65% B (25–30 min); 65–75% B (30–40 min); 75–90% B (40–50 min); 90–10% B (50–55 min). A photodiode array detector (PDA) was carried out for detection, by setting the wavelength to 280 nm. The polyphenols were characterized via co-injection, by comparing the retention times and UV data with standards. The analysis was realized in triplicate. To identify and quantify of the polyphenols, a calibration curve was obtained via the known concentrations of standard compound injections (0.0, 0.00782, 0.01563, 0.03125, 0.0625, 0.125, 0.25, 0.5, and 1.0 ppm). A total of 26 phenolic compounds were identified, namely protocatechuic gallic acid, chlorogenic, *p*-hydroxybenzoic, vanillic, 3-hydroxybenzoic, syringic, ferulic, *p*-coumaric, rosmarinic, ellagic and trans-cinnamic acid, pyrocatechol, vanillin, catechin, 6,7-dihydroxy coumarin, coumarin, quercetin, rutin, luteolin, hesperetin, taxifolin, myricetin, apigenin, kaempferol, and chrysin. The results are presented in μg per g of dry weight.

### 4.4. Microbial Strains Used and Minimum Inhibitory Concentration (MIC) Determination

In this study, the microorganisms that was used are *Chromobacterium violaceum* CV12472, *Pseudomonas aeruginosa* PA01, and *Chromobacterium violaceum* CV026. The microtiter broth dilution method was used for MIC determination as recommended by the Clinical and Laboratory Standards Institute [63]. The lowest plant extract concentration that yielded no visible growth was defined as MIC. Mueller–Hinton Broth (MHB) was the test medium and the 5 × 105 colony-forming units (CFU)/mL was the density of bacteria. A total of 100 μL of cell suspensions were inoculated into wells (96-well microtiter plates) together with the different final concentrations of the sample extracts (2, 1, 0.5, 0.25, 0.125, 0.0625, 0.03125 mg/mL) prepared in distilled water. The microplates were inoculated and incubated for 24 h at 37 °C before reading.

### 4.5. Violacein Inhibition Method Using C. violaceum CV12472

Firstly, to find the quorum-sensing inhibition (QSI) potentials against *C. violaceum* ATCC 12472, all the sample extracts were analyzed qualitatively [46]. A total of 10 µL of overnight culture of *C. violaceum* was adjusted to 0.4 OD at 600 nm and added into sterile microplates containing 200 µL of LB broth and was then incubated in the presence and in the absence of sub-MIC concentrations of the extracts prepared in distilled water. Broth with *C. violaceum* CV 12472 was used as a control positive. These plates were incubated for 24 h at 30 °C when a diminution production of violacein pigment was observed. The absorbance used for recording was 585 nm. Each experiment was realized in triplicate and the violacein inhibition percent was calculated using the following formula:

$$\mathrm{Violacein}\;\mathrm{inhibition}\;(\%)=\frac{OD\;585\;control-OD\;585\;sample\;}{OD\;585\;control}\;\times \;100$$

### 4.6. Quorum-Sensing Inhibition (QSI) Bioassay Using C. violaceum CV026

The evaluation of quorum-sensing inhibition was done as described elsewhere [47]. Firstly, 5 mL of warm molten Soft Top Agar (2.0 g Tryptone, 1.3 g Agar agar, 1.0 g sodium chloride in 200 mL deionized water) was seeded with CV026 overnight culture, 100 µL. C_6_HSL 20 µL was added as an exogenous AHL (Acyl Homoserine Lactone) source. This mixture was softly stirred and combined immediately over the surface of a solidified Luria Bertani Agar (LBA) plate as a film. A total of 5 mm in diameter of the wells were made after solidification of the overlay, on each plate. Each well was then filled with 50 µL of filter-sterilized extracts solution in distilled water at a sub-MIC concentration. QSI is indicated as a cream or white-colored halo around this well against a purple lawn of activated bacteria, CV026. The activity detection limit was made by employing serial dilutions of the extracts (from 1:1 to 1:8, using as diluent LB broth). The estimated endpoints were as the lowest dilution of sample extracts, leading to distinguishable inhibition of synthesis of violacein pigment. Each experiment was realized in triplicate. The microplates were incubated for three days at 30 °C, and then the quorum-sensing inhibition zones diameters were measured.

### 4.7. P. aeruginosa PA01 Swarming Motility Inhibition Assay

The swarming motility inhibition test was realized as previously described [64]. Shortly, *P. aeruginosa* PAO1 strain overnight cultures were point inoculated at the center of swarming plates containing 1% peptone, 0.5% agar, 0.5% NaCl, and 0.5% of filter-sterilized D-glucose with various sample extracts concentrations (50, 75, and 100 µg/mL) in an aqueous solution. The plates were after incubated in an upright position, at an appropriate temperature for 18 h and the control experiment was carried out in the same way but without extracts. By following the bacterial cells swarm fronts, the swarming migration was recorded.

### 4.8. Anticholinesterase Activity

Spectrophotometry was used to measure the anticholinesterase activity by the enzymatic inhibition of enzymes acetylcholinesterase and butyrylcholinesterase described elsewhere with slight modifications [30,65]. Briefly, sodium phosphate buffer, 130 μL (100 mM, pH 8.0), 10 μL of sample extract solution, which was dissolved in ethanol in different concentrations, and buffer enzyme solution (AChE or BChE, 20 μL) were combined and incubated at 25°C for 15 min, and after 20 μL of 0.5 mM DTNB was added (5,5′-Dithio-bis(2-nitrobenzoic) acid). The reaction was then initiated by the addition of acetylthiocholine iodide (20 μL, 0.71 mM), or butyrylthiocholine chloride (20 μL, 0.2 mM). The yellow 5-thio-2-nitrobenzoate anion formation in the reaction of DTNB with thiocholine, released by the enzymatic hydrolysis of butyrylthiocholine chloride or acetylthiocholine iodide, respectively, was monitored via spectrophotometry at wavelength of 412 nm, using a 96-well microplate reader. The results were expressed as the enzyme inhibition percentage (%) for 200 μg/mL extract concentration.

### 4.9. Anti-Tyrosinase Activity Assay

A spectrophotometric method according to [30] was used for determining the inhibitory activity of tyrosinase. Mushroom tyrosinase was the enzyme used, and L-DOPA was the reaction’s substrate. Sodium phosphate buffer (150 μL, 100 mM, pH 6.8) and different concentrations of sample solution (10 μL) dissolved in ethanol and 20 μL tyrosinase (in buffer solution) were mixed and incubated at 37 °C for 10 min, when 20 μL L-DOPA was added. The absorbances were measured after 10 min incubation (at 37 °C) in a 96-well microplate, at 475 nm. The data are expressed as percentage of enzymatic inhibition (%) at a concentration of 200 μg/mL.

### 4.10. α-Amylase Inhibitory Assay

The method described elsewhere [66], was used to evaluate *Clinipodium nepeta* extracts of the α-amylase inhibition properties. A total of 50 µL of sample solution dissolved in ethanol was added to 150 µL of a mixture prepared by adding 1.5 mg of soluble starch to 150 μL of buffer solution ((0.2 M, pH 6.8) containing 17 mM NaCl), followed by the addition of 10 µL of α-amylase enzyme (25 unit/mL). The obtained mixture was incubated for 30 min at 37 °C. Afterward, 20 μL of NaOH (2N) and 20 μL of a color reagent (3,5-dinitrosalisylic acid (44 μM) + potassium sodium tartrate tetrahydrate (106 μM) + NaOH (40 μM)) was added. After that, a second incubation for 20 min at 100 °C was performed. The absorbance for the resulting solutions was read at 540 nm, and the results are reported as IC_50_ values. The standard used for the comparison was acarbose.

### 4.11. Antioxidant 

For the evaluation of the antioxidant potential, six methods were employed. Spectrophotometric measurements were carried out using a 96-well microplate reader. Methanol was employed as a negative control. IC_50_ and A_0.50_ values represent the concentrations that provide 50% of inhibition. Both values extracted graphically were used to compare the efficiencies of extracts with standards. All experiments were performed in triplicate and recorded as the average of three values (mean ± standard deviation). Significant differences between means were determined using Student’s test and *p* values < 0.05 were noticed as significant.

#### 4.11.1. ABTS^•+^ Radical Scavenging Assay

For this assay, a spectrophotometric method reported in literature was followed [67]. The radical solution was prepared by mixing ABTS (2,2′-azino-bis(3-ethylbenzothiazoline-6-sulfonic acid)) with potassium persulfate (7 mM and 2.45 mM, respectively). The resulting solution was kept in the shade at room temperature, between 12 and 16 h before its use. 40 µL of the tested solution (extract or standard) in methanol with various concentrations, were added to 160 µL of ABTS^•+^, then incubated for 10 min. Absorbance values were measured at 734 nm. The obtained results (Table 2) expressed as IC_50_ were compared with employed antioxidant standards (BHA and BHT).

#### 4.11.2. DPPH^●^ Radical Scavenging Activity

DPPH^●^ radical scavenging according to the method described in the literature [68], was performed to evaluate the antioxidant potential of different concentrations of investigated extracts. A total of 40 µL of the tested solution (extract or standard) in methanol was added to 160 µL of DPPH^●^ solution (0.06 mg dissolved in one mL of methanol and having an absorbance of 0.5 to 517 nm). The absorbance of all samples was measured at 517 nm. The antioxidant potential was deduced and estimated by comparing the results expressed as IC_50_ with BHA (butylated hydroxyanisole) as a positive control.

#### 4.11.3. Galvinoxyl Radical (GOR) Scavenging Assay

Galvinoxyl radical scavenging activity protocol [69], was elaborated to determine the inhibition capacity of *Clinipodium nepeta* extracts. A volume of 40 μL of different dilutions of samples solution (plant extracts or standard) in methanol was added to 160 μL of galvinoxyl (0.1 mM in methanol). After 120 min of light-free incubation at room temperature, absorbance values were read at 428 nm. To report the results, we relied on IC_50_ values compared with both values of BHA and BHT.

#### 4.11.4. Cupric Reducing Antioxidant Capacity (CUPRAC)

According to the CUPRAC method [70], with slight modifications, where a volume of 50 µL of CuCl2 (10 mM) was added to a mixture of 60 µL of NH_4_CH_3_CO_2_ (pH = 7.0 buffer, 1 M) and 40 µL of each sample solution (extract or standard) in methanol, followed by the addition of 50 µL of Neocupronin (7.5 mM). Absorbance values (at 450 nm) were collected after 60 min of incubation. The results reported as A_0.50_ values are compared with antioxidant positive controls (BHA and BHT).

#### 4.11.5. Phenanthroline Method

The phenanthroline method was performed as described elsewhere [71]. A total of 50 μL of FeCl_3_ (0.2%) was mixed with 10 μL of various dilutions of sample solutions (extract or standard) in methanol, followed by the addition of 30 μL of O-phenanthroline (0.5%) and adjusted with 110 μL of methanol. After 20 min of incubation at 30 °C, the absorbance was measured at 510 nm. The obtained results were expressed as A_0.50_ values and compared with the used antioxidant positive controls (BHA and BHT).

#### 4.11.6. Ferric Reducing Antioxidant Power Assay

FRAP assay of *Clinopodium nepeta* extracts were evaluated using the method of reducing power described previously [72]. A total of 40 µL of phosphate buffer (pH = 6.6; 0.2 M) was added to 10 µL of sample solution (extract or standard) in methanol, with various concentrations, followed by the addition of 50 µL of potassium ferricyanide (1%). The mixture was incubated for 20 min at 50 °C. Afterward, 50 µL of trichloroacetic acid solution (10%), 40 µL of distilled water, and 10 µL of ferric chloride solution (0.1%), were added successively, and the absorbance was measured at 700 nm. The obtained results were expressed as A_0.50_ values and compared with ascorbic and α-tocopherol.

### 4.12. Statistical Analysis

Each activity was done in triplicate. The results were recorded as means ± standard error of the mean. Student’s test was used to determine the significant differences between means and *p* < 0.05 were regarded as significant.

## 5. Conclusions

Phenolic compounds remain a meaningful class of bioactive compounds. Preliminary phenolic characterization of *C. nepeta* using HPLC-DAD indicates the presence of selected phenolic compounds. Studies on *C. nepeta* are scarce and, in this study, DCM, AcOEt, and BuOH extracts were prepared from this plant. The preliminary phenolic composition of the extracts determined by HPLC-DAD indicated the highest number of phenolic compounds in the BuOH extract suggesting that phenolic composition could depend on the polarity of the solvent of extraction. These extracts showed anti-quorum-sensing activity in *C. violaceum* CV026 and inhibited violacein production in *C. violaceum* CV12472. Quorum sensing involves communication networks used by bacteria to increase severity and resistance against antibiotics. The extracts equally inhibited key enzymes such as AChE, BChE, tyrosinase, and α-amylase. The moderate to good enzyme inhibition by *C. nepeta* extracts indicates that they can be used in managing Alzheimer’s disease and diabetes, amongst others. Appreciable antioxidant activities were observed for the extracts and some of the phenolic compounds detected in them are known to have antioxidant effects. This suggests that the antioxidant effects of *C. nepeta* extracts may be attributed to the phenolic compounds detected in them. Natural plant extracts have significance because reactive oxygen species are at the origin of many ailments causing oxidative damage to tissues and it is necessary to prevent this by using antioxidants especially those from natural origin. The interest in natural and non-harmful medicinal plants lies in their usefulness and therapeutic alternatives to various ailments and the potential bioactivities exhibited by *C. nepeta* is good indication and justification of its application in traditional medicine.

## Figures and Tables

**Table 1:** Composition of *C nepeta* extracts in phenolic compounds determined by HPLC-DAD (µg/g).

No	Phenolic Compounds	RT (Retention Time, (min))	DCM Extract	AcOEt Extract	BuOH Extract
1	Gallic acid	5.70	3.21 ± 0.35	2.88 ± 0.11	3.41 ± 0.22
2	Protocatechuic acid	8.85	-	8.24 ± 0.25	8.83 ± 0.15
3	Catechin	10.68	-	-	-
4	Pyrocatechol	11.04	-	-	-
5	Chlorogenic acid	12.35	-	18.79 ± 0.17	30.56 ± 0.34
6	*p*-Hydroxy benzoic acid	12.77	-	14.48 ± 0.26	16.25 ± 0.13
7	6.7-Dihydroxy coumarin	14.10	-	-	-
8	Vanillic acid	15.18	2.18 ± 0.27	5.64 ± 0.10	4.83 ± 0.18
9	3- Hydroxy benzoic acid	15.98	-	-	-
10	Syringic acid	16.56	-	1.50 ± 0.12	2.33 ± 0.10
11	Vanillin	17.78	7.37 ± 0.11	1.34 ± 0.10	2.27 ± 0.14
12	*p*-Coumaric acid	20.56	-	-	-
13	Taxifolin	21.26	-	-	-
14	Ferulic acid	22.14	-	-	-
15	Coumarin	24.49	-	-	10.45 ± 0.23
16	Rutin	25.30	-	5.83 ± 0.18	12.20 ± 0.25
17	Ellagic acid	26.11	-	-	-
18	Rosmarinic acid	26.77	3.05 ± 0.12	25.91 ± 0.22	88.51 ± 0.55
19	Myricetin	27.35	-	72.58 ± 0.57	52.40 ± 0.36
20	Quercetin	30.83	6.43 ± 0.20	10.33 ± 0.15	6.83 ± 0.27
21	*trans*-Cinnamic acid	31.34	4.21 ± 0.35	5.28 ± 0.11	-
22	Luteolin	31.70	7.54 ± 0.20	8.31 ± 0.17	5.21 ± 0.12
23	Hesperetin	32.14	8.95 ± 0.28	9.27 ± 0.10	5.57 ± 0.20
24	Kaempferol	33.21	-	-	7.40 ± 0.18
25	Apigenin	33.57	21.75 ± 0.41	12.63 ± 0.24	4.84 ± 0.11
26	Chrysin	38.43	-	-	-

Values expressed are means ± S.E.M. of three simultaneously measurements (*p* < 0.05). -: not detected.

**Table 2:** Violacein inhibition percentage and quorum-sensing inhibition (diameter zones) of *C. nepeta* extracts.

Extracts	*C. violaceum* CV12472			*C. violaceum* CV026		
	DCM Extract	AcOEt Extract	BuOH Extract	DCM Extract	AcOEt Extract	BuOH Extract
MIC (mg/mL)	0.5	1	1	0.5	0.625	0.625
	Violacein Inhibition (% inh.)			QS Inhibition Zones (mm)		
MIC	100.0 ± 0.0	18.9 ± 4.1	-	10.3 ± 0.8	12.0 ± 0.5	-
MIC/2	19.2 ± 1.1	4.5 ± 0.3	-	-	-	-
MIC/4	9.0 ± 0.5	-	-	-	-	-
MIC/8	-	-	-	-	-	-

Values represent the means ± SEM of three simultaneously measurements (*p* < 0.05). -: no inhibition.

**Table 3:** Anti-swarming activities of *C. nepeta* extracts on *P. aeruginosa* PA01.

	Swarming Inhibition (% inh.)		
	100 µg/mL	75 µg/mL	50 µg/mL
DCM extract	14.89 ± 0.40	-	-
AcOEt extract	35.42 ± 1.00	17.95 ± 2.01	7.60 ± 0.33
BuOH extract	08.27 ± 3.11	-	-

Values represent the means ± SEM of three simultaneously measurements (*p* < 0.05). -: no inhibition.

**Table 4:** Enzyme (cholinesterase, tyrosinase, α-amylase) inhibitory activities of *C. nepeta* extracts.

	Cholinesterase Inhibitory Activity							
	AChE Assay		BChE Assay		Tyrosinase Inhibitory Activity		α-Amylase Inhibitory Assay	
	Inhibition (%) (at 200 µg/mL)	IC_50_ (µg/mL)	Inhibition (%) (at 200 µg/mL)	IC_50_ (µg/mL)	Inhibition (%) (at 200 µg/mL)	IC_50_ (µg/mL)	Inhibition (%) (at 200 µg/mL)	IC_50_ (µg/mL)
DCM extract	56.54 ± 0.61	170.1 ± 1.58	80.05 ± 0.69	73.06 ± 0.83	8.42 ± 0.34	>200	36.60 ± 1.82	>200
AcOEt extract	29.40 ± 0.29	>200	52.65 ± 0.96	187.8 ± 1.57	35.71 ± 0.91	>200	17.68 ± 2.52	>200
BuOH extract	12.31 ± 0.78	>200	30.77 ± 0.45	>200	14.78 ± 0.55	>200	17.21 ± 1.37	>200
Galantamine	83.43 ± 0.67	5.50 ± 0.18	76.51 ± 0.31	42.38 ± 0.50	NT	NT	NT	NT
Kojic acid	NT	NT	NT	NT	75.27 ± 0.56	23.80 ± 0.25	NT	NT
Acarbose	NT	NT	NT	NT	NT	NT	37.21 ± 3.54	365.93 ± 10.70

Values represent the means ± SEM of three simultaneously sample measurements (*p* < 0.05). NT: not tested. Inh. (%): percentage inhibition.

**Table 5:** Antioxidant activity of *C. nepeta* extracts.

	DPPH^●^ Assay	ABTS^●^_+_ Assay	GOR Assay	CUPRAC Assay	Phenanthroline Assay	FRAP Assay
Test sample	IC_50_ µg/mL	IC_50_ µg/mL	IC_50_ µg/mL	A_0.50_ µg/mL	A_0.50_ µg/mL	A_0.50_ µg/mL
DCM extract	42.77 ± 0.57	9.56 ± 1.12	32.05 ± 0.24	56.25 ± 1.08	21.46 ± 0.08	27.06 ± 1.49
AcOEt extract	26.98 ± 0.10	21.04 ± 0.83	21.75 ± 0.64	38.83 ± 0.93	10.72 ± 0.06	21.87 ± 0.89
BuOH extract	8.12 ± 0.11	12.82 ± 2.62	10.07 ± 0.40	29.44 ± 0.65	9.85 ± 0.07	17.42 ± 0.25
BHA	5.73 ± 0.41	1.03 ± 0.00	5.38 ±0.06	3.64 ± 0.19	0.93 ± 0.07	NT
BHT	NT	1.59 ± 0.03	3.32 ± 0.18	9.62 ± 0.87	2.24 ± 0.17	NT
α-Tocopherol	NT	NT	NT	NT	NT	34.93 ± 2.38
Ascorbic acid	NT	NT	NT	NT	NT	6.77 ± 1.15

Values expressed are means ± S.E.M. of three simultaneously measurements (*p* < 0.05). NT: not tested.
